# Combination of MiR-378 and MiR-210 Serum Levels Enables Sensitive Detection of Renal Cell Carcinoma

**DOI:** 10.3390/ijms161023382

**Published:** 2015-09-29

**Authors:** Michal Fedorko, Michal Stanik, Robert Iliev, Martina Redova-Lojova, Tana Machackova, Marek Svoboda, Dalibor Pacik, Jan Dolezel, Ondrej Slaby

**Affiliations:** 1 Department of Urology, University Hospital Brno and Masaryk University Brno, Brno 62500, Czech Republic; E-Mails: michal.fedorko@fnbrno.cz (M.F.); dalibor.pacik@gmail.com (D.P.); 2 Department of Urologic Oncology, Masaryk Memorial Cancer Institute, Brno 65653, Czech Republic; E-Mails: stanik@mou.cz (M.S.); dolezel@mou.cz (J.D.); 3 Department of Comprehensive Cancer Care, Masaryk Memorial Cancer Institute, Brno 65653, Czech Republic; E-Mails: 269069@mail.muni.cz (R.I.); msvoboda@mou.cz (M.S.); 4 Central European Institute of Technology, Masaryk University, Brno 62500, Czech Republic; E-Mails: martina.redova@gmail.com (M.R.-L.); tana.machackova@gmail.com (T.M.)

**Keywords:** renal cell carcinoma, microRNA, blood serum, biomarker

## Abstract

Serum microRNAs are emerging as a clinically useful tool for early and non-invasive detection of various cancer types including renal cell carcinoma (RCC). Based on our previous results, we performed the study to analyze circulating serum miR-378 and miR-210 in patients with various histological subtypes of RCC. RNA was purified from blood serum samples of 195 RCC patients and 100 healthy controls. The levels of miR-378 and miR-210 in serum were determined absolutely using quantitative real-time PCR. Pre- and postoperative levels of both microRNAs were compared in 20 RCC patients. Significantly increased serum levels of both miR-378 and miR-210 enabled to clearly distinguish RCC patients and healthy controls with 80% sensitivity and 78% specificity if analyzed in combination (*p* < 0.0001), and their levels significantly decreased in the time period of three months after radical nephrectomy (*p* < 0.0001). Increased level of miR-378 positively correlates with disease-free survival (*p* = 0.036) and clinical stage (*p* = 0.0476). The analysis of serum miR-378 and miR-210 proved their potential to serve as powerful non-invasive diagnostic and prognostic biomarkers in RCC.

## 1. Introduction

Renal cell carcinoma (RCC) is the most common neoplasm of adult kidney accounting for about 3% of adult malignancies with the mortality rate of over 40% [1]. There are several RCC subtypes, which can be further differentiated based on histological features including (i) clear cell RCC (conventional, 70%–80%); (ii) papillary RCC (10%–15%); and (iii) chromophobe RCC (5%) as the most common variants [2]. Demographically, the incidence rates are the highest in Europe, North America, and Australia, with Czech Republic having the highest incidence rate worldwide. Although there is a decreasing incidence of advanced stages of RCC (stage migration) in European cohorts in the last 25 years [3,4], the numbers of RCC patients diagnosed with advanced disease is still significant. As the survival rates for patients with localized disease compared with patients with regional and distant metastasis are significantly better, it is of great interest to provide early detection and treatment. Unfortunately, there is no standard serum biomarker enabling early and non-invasive diagnosis or monitoring of the disease. Recent reports highlighted the potential of serum microRNAs (miRNAs) to serve as a suitable tool for improving the RCC management.

MiRNAs are non-protein-coding, 18–25 nt in length, small RNAs involved in essential biological processes by their ability to regulate gene expression in a post-transcriptional manner. There is a growing number of evidence busting the myth of miRNAs being strictly intracellular molecules, confirming miRNAs in various body fluids, *i.e.*, serum/plasma, urine, and other body fluids in a highly stable form with similar signatures in men and women, as well as individuals of different age [5]. Although the existence of miRNAs in circulation and their originating from (i) microvesicles (released by exocytosis); (ii) exosomes (released upon fusion of late endosome with plasma membrane); or (iii) apoptotic vesicles and/or senescent bodies still remains to be clearly described and elucidated [6], circulating miRNAs constitute an elegant tool for early detection of RCC.

In our study, we analyzed circulating serum miRNAs (miR-378 and miR-210, formerly studied in both tissue and serum) [7,8,9] in patients with various histological subtypes of RCC by absolute quantification with respect to relevant clinical-pathological features confirming their potential to serve as a powerful non-invasive diagnostic and prognostic biomarker in RCC.

## 2. Results

**Figure 1:**
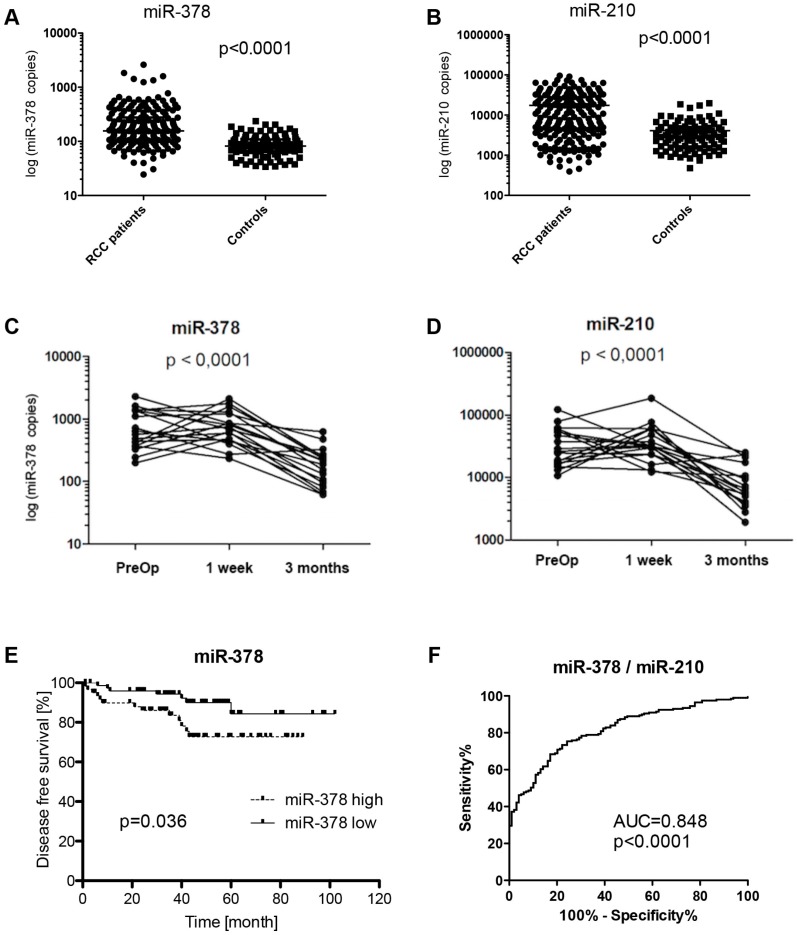
MiR-378 and miR-210 as biomarkers in renal cell carcinoma. Differences in serum levels of miR-378 (**A**) and miR-210 (**B**) in RCC patients and healthy controls; dynamics of miR-378 (**C**) and miR-210 (**D**) levels in blood serum one week and three months after radical nephrectomy; and positive correlation of miR-378 with disease free survival (**E**) and ROC analysis of miR-378/miR-210 combination for discrimination of renal cell carcinoma patients and healthy controls (**F**).

The serum expression levels of both miR-378 and miR-210 were significantly increased in RCC patients (*n* = 195) compared to healthy donors (*n* = 100) (*p* < 0.0001 for both) (Figure 1A,B), confirming our previous results with miR-378 on an independent cohort of RCC patients. Receiver operating characteristic (ROC) curve analysis revealed that the serum levels of both analyzed miRNAs could serve as appropriate biomarkers for differentiating serum of RCC patients from healthy controls with the area under curve (AUC) of 0.82 (95% CI, 0.77 to 0.86) for miR-378, and 0.74 (95% CI, 0.69 to 0.80) for miR-210, respectively (Table 1), even in the case when RCC patients with early clinical stages (stage I/II) were evaluated separately (*p* < 0.0001). Moreover, the combination of miR-378 and miR-210 could further improve the diagnostic accuracy with AUC of 0.85 (95% CI, 0.81 to 0.89, *p* < 0.0001) reaching the 80% sensitivity and 78% specificity (Figure 1F).

When miR-378 and miR-210 serum levels were analyzed one week and three months after radical nephrectomy, expression levels of both miRNAs were significantly decreased in the time period of three months (*p* < 0.0001) (Figure 1C,D).

**Table 1:** Summary of expression levels of miR-378 and miR-210 detected in serum of RCC patients and healthy controls expressed as median and interquartile range of miRNA copies.

Clinical Characteristic	*n*	MiR-378	MiR-210
RCC patients *vs.* healthy controls			
RCC	195	159 (109–278)	8544 (3344–27,269)
HC	100	83 (66–110)	2921 (1527–4953)
*p*-value		*p* < 0.0001	*p* < 0.0001
AUC		0.82	0.74
Histological subtype			
clear cell RCC	157	155 (109–270)	8111 (3288–26,929)
chromophobe RCC	12	201 (142–364)	9731 (3253–30,338)
papillary RCC	26	194 (88–281)	7760 (4551–23,223)
*p*-value		*p* = 0.4200	*p* = 0.9999
Clinical stage			
I	106	138 (104–238)	8775 (4385–25,151)
II	27	167 (113–230)	5000 (1904–17,732)
III	26	200 (152–268)	9078 (1561–27,439)
IV	36	226 (124–485)	14,909 (2683–36,245)
*p*-value		*p* = 0.0476	*p* = 0.3985
Fuhrman grade			
G1	34	141 (119–217)	8706 (3790–24,693)
G2	81	154 (102–287)	9999 (4151–32,338)
G3	55	159 (109–273)	7136 (2727–25,998)
G4	17	221 (167–501)	7286 (2357–15,398)
not available	8		
*p*-value		*p* = 0.1925	*p* = 0.7516

We also analyzed whether miR-378 and miR-210 serum expression levels were correlated to common clinical-pathological features of RCC. We observed a correlation between elevated serum miR-378 expression level and clinical stage (*p* = 0.0476), and miR-378 expression level and disease free survival (*p* = 0.036) (Figure 1E). We performed also the multivariate analysis of miR-378 together with common prognostic factors in RCC like T-, N-stage and Fuhrmann grade. Unfortunately, only T3 (*p* = 0.001) and N2 (*p* = 0.0006) stages were identified as independent prognostic factors. Circulating miR-378 has not reached statistical significance as independent prognostic factor in RCC (*p* = 0.1672).

However, neither serum miR-378 nor miR-210 levels were correlated with Fuhrman grade (*p* = 0.1925 for miR-378, *p* = 0.7516 for miR-210), and overall survival. We have not observed any difference in miRNA levels among RCC histological subtypes (*p* = 0.4200 for miR-378, *p* = 0.9999 for miR-210).

## 3. Discussion

Regarding the emerging evidence of circulating miRNAs to serve as relevant non-invasive biomarkers in cancer patients (e.g., miR-141 and miR-26a in prostate cancer [10,11], miR-29a and miR-92 in colorectal cancer [12], miR-195 in breast cancer [13], and based on our previous studies regarding the miR-378 and miR-210 potential to serve as an accurate biomarker both in circulating and tissue manner, we further evaluated these miRNAs in serum in the independent cohort of RCC patients undergoing radical nephrectomy (*n* = 195) and healthy donors (*n* = 100) using qRT-PCR. The present study showed that both serum miR-378 and miR-210 expression levels were significantly higher in RCC patients enabling clear distinguishing between RCC patients and healthy controls, even in early stages, and that their combination could serve as a powerful diagnostic biomarker with high accuracy (AUC 0.8480, 80% sensitivity, 78% specificity). The increased serum levels of miR-378 and miR-210 were observed also in the studies of Hauser *et al.* (2012), who described elevated miR-378 level in 25 ccRCC patients (*p* = 0.006), but were not able to distinguish between larger cohort of RCC with various histology and healthy controls [14], and Zhao *et al.* (2013) who observed significantly higher levels of miR-210 in the serum of 68 ccRCC patients (*p* < 0.001) [15]. Moreover, one of the typical and most prominent features of RCC tumors is hypoxia, and miR-210 is one of the well described so-called hypoxi-miRs [8]. We observed no changes in miR-378 and miR-210 serum levels one week after radical nephrectomy, probably due to pro-longed half-lives of both miR-378 and miR-210. However, in the time period of three months after surgery expression levels of both miRNAs significantly decreased (*p* < 0.0001), which was observed also by Zanutto *et al.* (2014) in colorectal cancer [16]. For the first time, we have described association of elevated serum levels of miR-378 with clinical stage (*p* = 0.0476) and disease-free survival (*p* = 0.036), extending also the results of our previous study [7]. Unfortunately, we were not able to prove circulating miR-378 as an independent prognostic factor in RCC by multivariate analysis.

Our study has several limitations, which should be discussed. There was a study published showing superiority of plasma over serum for circulating miRNAs analysis based on the release of platelets or WBC miRNA contents to the serum during the coagulation process [17]. Our measurement should not be biased from this perspective, because miR-378 and miR-210 were not shown to be associated with platelet or WBCs. Another limitation is definitely absolute quantification approach, which we have used for determination of studied miRNAs disabling to eliminate methodical inaccuracies, which could occur in processing of every sample and, finally, could bias comparisons of different groups of samples. However, we believe that the studied groups of patients and controls are large enough to overcome this bias and, mainly, there is no conclusively-defined reference gene, which should be used for normalization of circulating miRNAs expression.

In conclusion, serum miR-378 and miR-210 (or their combination) could serve as a powerful diagnostic and even prognostic biomarker in management of RCC patients.

## 4. Methods

### 4.1. Study Population

Serum samples from RCC patients were collected at the Masaryk Memorial Cancer Institute (MMCI; Brno, Czech Republic) and University Hospital Brno (UHB; Brno, Czech Republic). The study group included patients diagnosed for RCC and undergoing radical nephrectomy at MMCI (*n* = 195; male: 133, female: 62, median age: 64), cancer-free blood donor volunteers (*n* = 100; male: 65, female: 35, median age: 52) recruited from Department of Transfusion and Tissue Medicine of UHB, with no history of any type of cancer, and twenty RCC patients from UHB with serum samples additionally collected one week and three months after nephrectomy. Both cancer patients and healthy controls were of the same ethnicity (Caucasian). Clinical and pathological characteristics including stage and grade are summarized in Table 1. RCC serum samples were collected after signing an informed consent prior to surgery and stored at MMCI Bank of Biological Material. The study (MZ09-MOU-SlabyOndrej-B) has been approved by ethical committee of MMCI (3 September 2008).

### 4.2. RNA Isolation

Blood serum samples were collected just before surgery and initiation of any oncological treatment. Blood was processed for serum within one hour after extraction. Serum was stored in liquid nitrogen and the median of storage time to endpoint analysis was 20 months. Serum was obtained by centrifugation at 1200× *g* for 10 min at 4 °C. To complete the removal of residual cellular components, serum samples were re-centrifuged at 12,000× *g* for a further 10 min at 4 °C. Total RNA enriched for small RNAs was isolated using Qiagen miRNeasy Mini Kit (Qiagen, GmbH, Hilden, Germany) from 250 µL of blood serum according to modified manufacturers’ protocol (we added 1.25 µL of MS2 RNA (0.8 µg/µL) to QIAzol (Qiagen, GmbH, Hilden, Germany). Concentration and purity of RNA were determined by measuring its optical density (A260/280 > 2.0; A260/230 > 1.8) using NanoDrop ND-1000 Spectrophotometer (Thermo Scientific, Wilmington, DE, USA). The samples were either stored at −80 °C or further processed.

### 4.3. qRT-PCR Quantification of MiRNA Expression in Serum

MiR-378 and miR-210 were quantified by TaqMan MicroRNA Assays (Applied Biosystems, Carlsbad, CA, USA) following reverse transcription (TaqMan MicroRNA Reverse Transcription Kit, Applied Biosystems) of 3 µL of RNA on Applied Biosystems 7500 instrument following the manufacturers’ protocols. To reduce the possible high intra-assay variance introduced by low abundant miRNA, a pre-amplification step using TaqMan PreAmp Master Mix (Applied Biosystems) was performed for serum RNA samples prior to miR-210 analysis according to the manufacturer’s instructions. Absolute quantification of miRNAs was performed in triplicate.

### 4.4. Statistics

Statistical analyses were performed using GraphPad Prism version 6 (GraphPad software, La Jolla, CA, USA). Sensitivity, specificity and area under curve (AUC) for miRNA levels were determined using Receiver Operator Characteristic (ROC) analysis. Clinical-pathological parameters and miRNA levels were correlated using the Mann–Whitney-U or Kruskal–Wallis-test, as appropriate. Kaplan–Meier survival curves and long-rank test were used for survival analysis.

## 5. Conclusions

There are significantly increased levels of both miR-378 and miR-210 in blood serum of RCC patients when compared to age and gender-matched healthy donors. These miRNAs enable to clearly distinguish RCC patients and healthy controls with 80% sensitivity and 78% specificity if analyzed in combination, and their levels significantly decrease in the time period of three months after radical nephrectomy. Moreover, increased levels of miR-378 correlates with disease-free survival and clinical stage in RCC patients. In conclusion, analysis of miR-378 and miR-210 serum levels proved their potential to serve as powerful non-invasive diagnostic and prognostic biomarkers in RCC.
